# Arginase II inhibitory activity of flavonoid compounds from *Scutellaria indica*

**DOI:** 10.1007/s12272-013-0125-3

**Published:** 2013-04-20

**Authors:** Sang Won Kim, To Dao Cuong, Tran Manh Hung, Sungwoo Ryoo, Jeong Hyung Lee, Byung Sun Min

**Affiliations:** 1 College of Pharmacy, Catholic University of Daegu, Gyeongsan, Gyeongbuk 712-702 Republic of Korea; 2 College of Natural Science, Kangwon National University, Chuncheon, Kangwon 200-701 Republic of Korea

**Keywords:** *Scutellaria indica*, Labiatae, Flavonoid, Arginase II inhibitory activity

## Abstract

Arginase II has recently reported as a novel therapeutic target for the treatment of cardiovascular diseases such as atherosclerosis. In the course of screening plants used in natural medicines as arginase II inhibitory activity, a methanol extract of *Scutellaria indica* showed significant inhibitory effect. Further fractionation and repeated column chromatography led to the isolation of a new flavan-type (**1**), and seven known compounds (**2**–**8**). The chemical structures of isolated compounds were elucidated based on extensive 1D and 2D NMR spectroscopic data. The isolates **1**–**8** were investigated in vitro for their arginase II inhibitory activity using enzyme solution prepared from kidney of anesthetized C57BL/6 mice. Compounds **3** and **5** significantly inhibited arginase II activity with IC_50_ values of 25.1 and 11.6 μM, respectively, whereas the other compounds were apparently inactive.

## Introduction

Arginine is the common substrate for both arginase and nitric oxide synthase (NOS). Arginase hydrolyses arginine to ornithine and urea, whereas NOS converts arginine to nitric oxide (NO) and citrulline. In the wound and elsewhere, arginase expression regulates arginine bioavailability for NO synthesis. Arginase is present in two isoforms as arginase I, the hepatic isoform, and arginase II, the extrahepatic isoform, each of which is encoded by a distinct gene. Arginase activation/upregulation results in arginase/NOS imbalance, decrease NO production, and contributes to endothelial dysfunction in a number of pathophysiological processes such as aging (Berkowitz et al. 2003) diabetes (Bivalaqua et al. 2001), hypertension (Demougeot et al. 2005; Johnson et al. 2005) and atherosclerosis (Ryoo et al. 2006, 2008). Additionally, arginase enhances production of reactive oxygen species by eNOS. Arginase inhibition in hypercholesterolemic (ApoE(−/−)) mice or arginase II deletion (ArgII(−/−)) mice restores endothelial vasorelaxant function, reduces vascular stiffness, and markedly reduces atherosclerotic plaque burden. Furthermore, arginase activation contributes to vascular changes including polyamine-dependent vascular smooth muscle cell proliferation and collagen synthesis. Collectively, arginase may play a key role in the prevention and treatment of atherosclerotic vascular disease (Ryoo et al. 2011a, b).

To search for new type of arginase II inhibitors, hundreds of plant extracts were screened for activity against arginase II, and a methanol extract of *S. indica* was found to inhibit arginase II. *S. indica* (Labiatae) is a small herb, with erect stems arising from a prostrate base, and usually 15–30 cm tall. The plant often has a main stem and several side branches. The whole herb of *S.* *indica*, known as “Han-xin-cao,” is used for treatment of hemoptysis, hematemesis, anticancer, and other disease in China and is distributed widely in Korea, China, Taiwan, Japan, and Southeast Asia (Chiang Su New Medical College 1977). The methanolic extraction of the root of *S.* *indica* was found to have potent cytotoxic activity against L1210 and HL60 cells in an earlier in vitro screening test (Bae et al. 1994). Bae et al. confirmed that an ether extract of *S.* *indica* with flavonoid compounds has potent inhibition of cytotoxic effects (Bae et al. 1994), but its chemical composition and biological evaluation have not yet been performed thoroughly in regard to the inflammatory activity of this plant. Therefore, to investigate inhibitors of arginase II, further fractionation of the EtOAc-soluble fraction resulted in the isolation of a new compound, along with seven known compounds. This study describes the isolation and structural elucidation of these isolates and their inhibitory arginase II activity.

## Materials and methods

### General experimental procedure

Optical rotation was measured with a JASCO DIP 370 digital polarimeter. UV spectra were obtained in MeOH using a Thermo 9423AQA2200E UV spectrometer, and IR spectra were obtained on a Bruker Equinox 55 FT-IR spectrometer. The nuclear magnetic resonance (NMR) spectra were obtained on varian unity inova 400 MHz spectrometer. ECD spectra were recorded on a JASCO J-810 spectropolarimeter. EI-MS and HR-EI-MS spectrometric data were acquired with a JMS-700 MSTATION mass spectrometer (JEOL, Japan). Silica gel (Merck, 63–200 μm particle size) and RP-18 silica gel (Merck, 75 μm particle size) were used for column chromatography. TLC was carried out using Merck silica gel 60 F_254_ and RP-18 F_254_ plates. HPLC was performed using a Waters 600 Controller system with a UV detector and an YMC Pak ODS-A column (20 × 250 mm, 5 μm particle size, YMC Co., Ltd., Japan) and HPLC solvents were from Burdick & Jackson, USA.

### Plant material

*S. indica* was collected in Jindo island, Korea, in May 2011. Botanical identification was performed by Prof. Byung-Sun Min, and the voucher specimen CUD-1523 was deposited at the herbarium of the College of Pharmacy, Catholic University of Daegu, Korea.

### Extraction and isolation

**Fig. 1:**
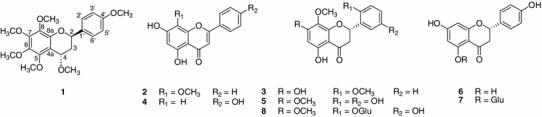
Chemical structure of isolated compounds **1**–**8**

The air-dried whole plant of *S. indica* (3.09 kg) was extracted with MeOH (15 L) at room temperature for 7 days and then MeOH extract (408.9 g) was suspended in hot-water (2 L) and partitioned with *n*-hexane (3 L × 3), ethyl acetate (3 L × 3), and *n*-butanol (3 L × 3), successively. The resulting fractions were concentrated in vacuo to give the hexane- (179.3 g), EtOAc- (62.0 g) and BuOH-soluble fraction (26.8 g), respectively. By the activity-guided fractionation, the EtOAc-soluble fraction (62.0 g) was applied to a silica gel column eluted with CHCl_3_–MeOH (50:1 to 0:1) to yielded 15 subfractions (E1 ~ E15). Subfraction E6 (1.85 g) was subjected on a silica gel column, eluted with hexane–acetone (15:1 to 5:1) to afford nine subfractions (E6.1 ~ E6.9). Compound **1** (14.3 mg) was crystallized from subfraction E6.2 (328.4 mg) with hexane–acetone (10:1). Subfraction E6.5 (508.4 mg) was applied on a reverse phase silicagel column and eluted MeOH–H_2_O (1:1 to 5:1) to afford four subfraction (E6.5.1 ~ 6.5.4). Subfractions E6.5.2 (125.0 mg) was subjected to HPLC using MeOH–H_2_O (1:1 ~ 4:1) to obtain compound **2** (20.2 mg). Subfraction E6.5.4 (114.0 mg) was subjected to HPLC using MeOH–H_2_O(1:1 ~ 3:1) to obtain compound **3** (35.0 mg). Subfraction E6.6 (1.3 g) subjected to MPLC using MeOH–H_2_O (2:1) to furnish compound **5** (151 mg). Subfraction E.9 (1.65 g) was applied to a reverse phase silica gel column and eluted with MeOH–H_2_O (1:1 to 5:1) to afford three subfractions (E9.1 ~ E9.5). Compounds **4** (8.3 mg) and **6** (151.0 mg) were isolated from subfraction E9.3 by using HPLC on a RP-18 column using MeOH–H_2_O (70 : 30 → 100 : 0). Subfractions E12 (7.22 g) was subjected on a silica gel column, eluted with CHCl_3_–MeOH (10:1 to 1:1) to afford nine subfractions (E12.1 ~ E12.9). Subfraction E.12.2 (158.8 mg) was further subjected to HPLC on a RP-18 column using MeOH–H_2_O (30 : 70 → 50 : 50) to obtain compounds **7** (3.6 mg) and **8** (5.7 mg), respectively (Fig. 1)

(2*R*,4*S*)-4,5,6,7,8,4′-hexamethoxylflavanone (**1**): white crystal; m.p: 218–220 °C; [*α*] +14.7 (*c* 0.42, MeOH); UV (CHCl_3_) *λ*_max_ nm: 209, 282; CD (*c* 0.15, MeOH): ∆*ε*_208_ (nm) +1.95; ∆*ε*_229_ (nm) -7.84; HR-EI-MS *m/z*: 390.1679 [M]^+^ (calcd. for C_21_H_26_O_7_); ^1^H NMR (400 MHz in CD_3_OD) and ^13^C NMR (100 MHz in CD_3_OD) spectroscopic data, see Table 1.

**Table 1:** ^1^H NMR (400 MHz, CD_3_OD) and ^13^C NMR (100 MHz, CD_3_OD) spectroscopic data for compound **1**

Position	**1**	
	*δ*_H_ (ppm)	*δ*_C_ (ppm)
2	5.07 (1H, dd, *J* = 2.4, 12.4 Hz)	74.5
3	2.37 (1H, dt, *J* = 2.4, 14.4 Hz)	35.1
	1.89 (1H, ddd, *J* = 2.4, 14.4, 12.4 Hz)	
4	4.56 (1H, t, *J* = 2.4 Hz)	69.9
4a		113.3
5		149.6
6		141.1
7		149.4
8		139.2
8a		146.5
1′		134.6
2′, 6′	7.42 (2H, d, *J* = 8.8 Hz)	128.9
3′, 5′	6.98 (2H, d, *J* = 8.8 Hz)	115.0
4′		161.2
4-OCH_3_	3.52 (3H, s)	56.6
5-OCH_3_	3.94 (3H, s)	62.1
6-OCH_3_	3.85 (3H, s)	61.8
7-OCH_3_	3.92 (3H, s)	62.0
8-OCH_3_	3.78 (3H, s)	61.7
4′-OCH_3_	3.83 (3H, s)	55.9

### Arginase II

Arginase II solution was prepared from kidney lysates of anesthetized C57BL/6 mice (White et al. 2006). Tissue lysates of kidney were prepared using lysis buffer (50 mM Tris–HCl, pH7.5, 0.1 mM EDTA and protease inhibitors) by homogenization at 4 °C followed by centrifugation for 20 min at 14,000×*g* at 4 °C. Briefly, aortic lysates were added to Tris–HCl. The hydrolysis reaction of l-arginine by Arg was performed by incubating the mixture containing activated Arg and was stopped by adding acid solution. For calorimetric determination of urea, α-isonitrosopropiophenone was added, and the mixture was heated at 100 °C for 45 min. After placing the sample in the dark for 10 min at room temperature, the urea concentration was determined spectrophotometrically by the absorbance at 550 nm (White et al. 2006).

## Results and discussion

The MeOH extract of *S. indica* was partitioned into *n*-hexane-, EtOAc-, and *n*-BuOH-soluble fractions and a H_2_O layer. Chromatographic purification of the EtOAc-soluble fraction led to the isolation of eight compounds. The structures of known compounds were identified as wogonin (**2**) (Li et al. 2009), (2*S*)-5,7-dihydroxy-8,2′-dimethoxyflavanone (**3**) (Tomimori et al. 1985), apigenin (**4**) (Loo et al. 1986), (2*S*)-5,2′,5′-trihydroxy-7,8-dimethoxyflavanone (**5**) (Miyaichi et al. 1987), naringenin (**6**), naringenin-5-*O*-*β*-d-glucopyranoside (**7**) (Ibrahim et al. 2003) and (2*S*)-5,5′-dihydroxy-7,8-dimethoxyflavanone-2′-*O*-*β*-d-glucopyranoside (**8**) (Botha et al. 1981) by comparing their physiochemical and spectroscopic data with those reported in the literature (Fig. 1).

**Fig. 2:**
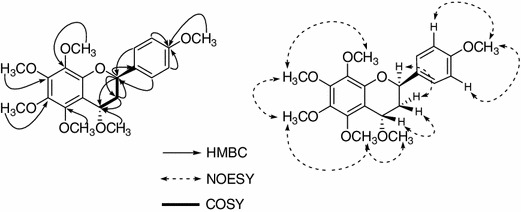
Selected HMBC, NOESY and COSY correlations of compound **1**

Compound **1** was isolated as a white crystal, with the molecular formula C_21_H_26_O_7_, as determined by the HR-EI-MS at *m*/*z* 390.1679 for the [M]^+^ ion (calculated for C_21_H_26_O_7_, 390.1678). The optical rotation value was +14.7, and the UV spectrum exhibited *λ*_max_ at 209 and 282 nm (MeOH). In accordance with the molecular formula, 21 carbon signals in the ^13^C-NMR spectrum of **1** were categorized as four *sp* ^2^ methines, eight *sp* ^2^ quaternary, six methoxyls, one *sp* ^3^ methylene and two *sp* ^3^ methines carbons. Apart from the six methoxyls, the remaining 15 carbons comprising its scaffold as one C6–C3–C6 unit with two benzene rings suggested that **1** was a flavanoid skeleton. The ^1^H-NMR spectrum of **1** showed six methoxyl signals at *δ*_H_ 3.52–3.94 (18H, s), and this information indicated that **1** has a hexasubstituted in two benzene rings. Furthermore, a typical AA′BB′ spin system at *δ*_H_ 7.42 (d, *J* = 8.8 Hz, H-2′,6′) and 6.98 (d, *J* = 8.8 Hz, H-3′,5′) was also observed. The characteristic signals of a flavan moiety as a AB_2_C coupling system at *δ*_H_ 5.07, 2.37, 1.89 and 4.56 were observed in the ^1^H-NMR spectrum of **1**, indicating that **1** might have a flavan part (Table 1). The presence of the flavan part was further confirmed by correlations of *δ*_H_ 5.07 (H-2) to *δ*_C_ 35.1 (H-3), 128.9 (C-2′, 6′) and 134.6 (C-1′); *δ*_H_ 4.56 (H-4) to *δ*_C_ 74.5 (C-2), 35.1 (C-3), 146.5 (C-8a), 113.3 (C4a) and 149.6 (C-5) in the HMBC experiment (Fig. 2). The positions of six methoxyl groups were determined based on the HMBC and NOESY spectra. In the HMBC spectrum, the long-range correlation between signals were observed at *δ*_H_ 3.83 (3H, s) and *δ*_C_ 161.2; *δ*_H_ 3.52 (3H, s) and *δ*_C_ 69.9; *δ*_H_ 3.94 (3H, s) and *δ*_C_ 149.6; *δ*_H_ 3.85 (3H, s) and *δ*_C_ 141.1; *δ*_H_ 3.92 (3H, s) and *δ*_C_ 149.4; *δ*_H_ 3.78 (3H, s) and *δ*_C_ 139.2. From the NOESY spectrum, the protons at *δ*_H_ 3.83 (3H, s) displayed NOEs with the aromatic proton signals at *δ*_H_ 6.98 (H-2′, 5′), and the methoxyl at *δ*_H_ 3.52 (3H, s) displayed NOEs with the methoxyl protons at *δ*_H_ 3.94 (3H, s). Furthermore, the methoxyl proton signal at *δ*_H_ 3.85 (3H, s) also displayed NOEs with the methoxyl proton signals at *δ*_H_ 3.94 (3H, s) and 3.92 (3H, s), and the methoxyl proton signal at *δ*_H_ 3.78 (3H, s) displayed NOEs with the methoxyl proton at *δ*_H_ 3.92 (3H, s), indicating that these methoxyl groups were located C-4′, C-4, C-5, C-6, C-7, and C-8. In addition, both H-2 and H-4 showed connectivity to H-3*a* and H-3*e* in the ^1^H–^1^H correlation spectroscopy (COSY) spectrum of compound **1** (Fig. 2).

The stereochemistry of compound **1** was defined on the basis of its nuclear Overhauser effect spectroscopy (NOESY) spectrum. NOE correlation was observed between H-2 and H-3*a*, while H-4 exhibited NOEs to H-3*e*. These results were in agreement with the relative stereochemistry, which was further confirmed by the coupling constant and split pattern. The coupling constants of the spin systems in the ^1^H NMR spectrum of **1** [*J*_2,3*a*_ = 12.4 Hz; *J*_2,3*e*_ = 2.4 Hz] indicated indicated H-2 retain as an axial bond (Botha et al. 1981). An *S* absolute configuration of compound **1** at C-4 was realized by a positive Cotton effect of ∆*ε*_208_ +1.95 and a negative Cotton effect of ∆*ε*_229_ -7.84 (Barrett et al. 1979). Therefore, it was presumed that compound **1** possessed a 2*R*,4*S* absolute configuration. Herein, the structure of compound **1** was established as (2*R*,4*S*)-4,5,6,7,8,4′-hexamethoxylflavan.

Arginase II activity is upregulated in atherosclerosis-prone mice, and is associated with impaired endothelial NO production, endothelial dysfunction, vascular stiffness, and ultimately, aortic plaque development. Conversely, inhibiting endothelial arginase or deleting the arginase II gene enhances NO production, restores endothelial function and aortic compliance, and reduces plaque burden. Therefore, arginase II represents a novel target for preventing and treating atherosclerotic vascular disease (Ryoo et al. 2008). In the present study, we screened the isolated compounds for anti-arginase II activity. The results showed that incubating compounds **3** and **5** from kidney lysates significantly inhibited arginase II activity with IC_50_ values of 25.1 and 11.6 μM, respectively, whereas the other compounds were apparently inactive (Table 2). In this study, piceatannol-3′-*O*-*β*-d-glucopyranoside (PG), was used as positive control (Woo et al. 2010), showed an IC_50_ value of 1.0 μM. Although the inhibitory potency of **3** and **5** against arginase II activity are higher compared to PG, the identification of new moiety from natural medicinal plants that inhibits arginase activity would be useful for the development of pharmaceutical natural compounds.

**Table 2:** Arginase II inhibitory activity of compounds **1**–**8**

Compounds	IC_50_ values (μM)^a^
**1**	>200
**2**	>200
**3**	25.1 ± 2.6
**4**	>200
**5**	11.6 ± 1.2
**6**	>200
**7**	>200
**8**	>200
**PG** ^b^	1.0 ± 0.1

^a^The inhibitory effects are represented as the molar concentration (μM) giving 50 % inhibition (IC_50_) relative to the vehicle control. These data represent the average values of three repeated experiments

^b^Piceatannol-3′-*O*-*β*-d-glucopyranoside (PG) was used as positive control
